# Premature release of action sequences in adolescent male rats

**DOI:** 10.3389/fnbeh.2026.1716850

**Published:** 2026-02-10

**Authors:** Micaela Betsabé Marini, Mariano Andrés Belluscio, Mario Gustavo Murer, María Cecilia Martínez

**Affiliations:** 1 Departamento de Fisiología, Biología Molecular y Celular “Dr. Héctor Maldonado”, Facultad de Ciencias Exactas y Naturales, Universidad de Buenos Aires, Buenos Aires, Argentina; 2 Laboratorio de Fisiología de Circuitos Neuronales, Grupo de Neurociencia de Sistemas, Instituto de Fisiología y Biofísica “Dr. Bernardo Houssay” (IFIBIO-Houssay), Universidad de Buenos Aires – CONICET, Buenos Aires, Argentina; 3 Departamento de Fisiología, Facultad de Ciencias Médicas, Universidad de Buenos Aires, Buenos Aires, Argentina

**Keywords:** adolescence, impulsivity, learning, reward, sex-differences

## Abstract

Adolescence is a period of transition from childhood to adulthood in which subjects exhibit characteristic behaviors, such as increases in impulsivity, social interactions, novelty-seeking and risk-taking behaviors. Previous studies have shown that adolescents often respond prematurely in operant tasks, but it remains unclear whether this behavior reflects general hyperactivity, cognitive impairments, or if it is a specific form of impulsivity. Here, we trained male and female adolescent and adult Long-Evans rats in a rewarded operant task that required withholding an action sequence for a 2.5 s waiting time. Adolescent males exhibited significantly more premature responses than females or adults, despite achieving similar reward rates. To test whether this behavior was associated with other traits, we assessed locomotor activity in the Open Field and recognition memory in the Y-maze. Locomotor activity and memory performance were comparable between groups, indicating that the increased premature responding observed in adolescent males was not readily explained by differences in general activity levels or cognitive performance. Together, these findings suggest that impulsive responding during adolescence shows a sex-dependent expression and reflects a distinct behavioral component within the context of this task, rather than belonging to broader behavioral differences. Understanding the specificity of adolescent impulsivity may provide insights into vulnerability to risk-taking and psychiatric disorders during adolescence.

## Introduction

Adolescence is a critical developmental stage characterized by significant physiological, neurobiological, and cognitive changes that prepare individuals for their independence in adult life. The profound transformations in the prefrontal cortex and limbic system shape the behavioral characteristics typically associated with it. In addition to these cortical and limbic changes, the striatal reward system also undergoes marked developmental remodeling during adolescence, contributing to heightened reward sensitivity and motivated behavior (Galvan, 2010; Naneix et al., 2012; Niemiec et al., 2026). There is an increase in impulsive, sensation-seeking, and risk-taking behaviors. Also, social interactions and validation by peers become particularly relevant in this period. Consequently, it is a period of extreme vulnerability to substance use disorders (Spear, 2000; Laviola et al., 2003; Jordan and Andersen, 2017). Furthermore, there is an increased sensitivity to stress, and it is the point where many psychiatric pathologies begin to emerge (Paus et al., 2008; Bava and Tapert, 2010; Hammerslag and Gulley, 2016; Andersen, 2019).

Impulsivity is a multidimensional construct present in humans and other mammals, often seen as a consequence of impaired inhibitory function. It is characterized by a predisposition for rapid actions without appropriate foresight, inefficient control of inhibitory responses, and alterations in how rewards are processed (Bari and Robbins, 2013; Voon, 2014; Kaiser et al., 2022). This premature responding, which is a key measure of impulsivity, can manifest as either a stable personality trait, an endophenotype associated with neuropsychiatric disorders, or a transient state related to substances, contexts, or stress. Although different aspects of impulsivity rely on partly dissociable neural mechanisms, they share overlapping cortico-striatal and hippocampal contributions (Dalley et al., 2011; Dalley and Robbins, 2017).

An influential view poses that there might be two types of impulsivity: impulsive action and impulsive choice. This taxonomy has been extensively reviewed; however, both components are interconnected and have been shown to predict different aspects of drug abuse (Weafer and de Wit, 2014; Dalley and Robbins, 2017). Impulsive action (or behavioral inhibition) refers to the difficulty in inhibiting or controlling one’s behavior. In contrast, impulsive choice involves the tendency to prefer smaller, immediate rewards over larger, delayed rewards (Weafer and de Wit, 2014). Impulsive choice is typically assessed using delay discounting tasks, where participants choose between receiving smaller rewards immediately or larger rewards that are available after a delay with higher certainty. Deficits in working memory have been linked to steeper discounting and greater impulsive choice in both rodents and humans (Panwar et al., 2014; Renda et al., 2014). In contrast, the inability to restrain a response in the 5-choice serial reaction time task (5CSRTT) depends on corticostriatal and subthalamic mechanisms (Voon, 2014; Dalley and Robbins, 2017). Although conceptually distinct, both forms of impulsivity relate to vulnerability to addiction and maladaptive decision-making (Weafer and de Wit, 2014).

During adolescence, impulsivity can manifest in multiple forms, including difficulties in inhibiting actions (impulsive action) and a tendency to favor immediate over delayed rewards (impulsive choice). These forms of impulsivity may co-occur with increased locomotor activity and suboptimal action planning (Romer, 2010; Romer et al., 2017). Previous works with rodents have shown that adolescents tend to exhibit more impulsive responses than adults, although the specific manifestations vary across tasks and contexts (Doremus-Fitzwater et al., 2012; Stolyarova and Izquierdo, 2015; Hammerslag and Gulley, 2016). Moreover, sex differences have been reported, with males often -though not always- showing greater impulsivity than females (Romer, 2010; Cross et al., 2011; Weinstein and Dannon, 2015; Hammerslag et al., 2019)

Here we assessed impulsive action using a rewarded task that requires the animal to inhibit premature responses before initiating a timed action sequence. With this paradigm, we have previously shown that adolescent males make significantly more impulsive responses compared to adults (Martinez et al., 2022). To evaluate whether premature responding reflects a general behavioral phenotype or a specific form of impulsivity, we complemented the rewarded task with assessments of locomotor activity (Open Field task) and recognition memory (Object Recognition in the Y-maze task). These measures were selected because locomotion can index general arousal or motor disinhibition, whereas recognition memory performance is relevant to cognitive processes linked to impulsive choice and working-memory-dependent decision-making.

Considering the differences in impulsiveness between males and females during adolescence (Romer, 2010; Weinstein and Dannon, 2015; Romer et al., 2017), we trained adolescents and adults of both groups in the rewarded task to characterize sex differences in action control and related behavioral domains.

## Materials and methods

### Subjects

The pups were weaned between postnatal day (P) 23 and p25, allowing several days for acclimation to pair housing and handling, and at least 1 day of water deprivation, which was initiated at that time and continued for 2 days before the beginning of behavioral training. Subjects were 16 adolescent and 16 adult Long-Evans rats from our colony (adolescent ∼30 days old (Ado)), 8 females (31–36 days old) and 8 males (28–36 days old), approximate weight ∼80–120 g at the beginning of the experiments; Adults (AD): ∼60 days old at the beginning of the experiments, 8 females (59–70 days old) and 8 males (60–72 days old), approximate weight ∼340–480 g), housed on a 12:12 light: dark cycle and experiments were performed during the light phase, 21° C room temperature. Littermates were assigned to the same age group (adolescent or adult), and, when possible, individuals from the same litter were distributed across experimental conditions to control for litter-related variability. Individual weights were not recorded, which we acknowledge as a limitation. Rats were housed in groups of 2 or 4 in regular cages with wood shavings as bedding and were identified by tail markings made with a marker pen, which was renewed when needed. Five days before the beginning of the experiments, animals were handled for 3 min (twice a day for 2 days) to minimize emotional stress. Two days before the beginning of the behavioral training subjects were deprived of water. Subjects were water-restricted to 20 min of access per day. Once per week, they received a rest period during which water was available *ad libitum* beginning 1 hour after the final training session of the week and continuing until the next deprivation day. For example, if the final session took place on Friday, subjects had unrestricted access to water from Friday evening until Sunday morning. This schedule maintained animals at 90% of their pre-deprivation weight, with any further weight loss being counteracted by increased free water access. All procedures complied with the National Institutes of Health Guide for Care and Use of Laboratory Animals (Publications No. 80-23, revised 1996) and were approved by the Animal Care and Use Committee of the University of Buenos Aires (CICUAL, RESCD-2022-315-E-UBA-DCT#FMED). In all paradigms, the same schedule was maintained for the training or testing sessions. Litter composition and the age of animals at the start of training and at each behavioral test (Open Field and Y-maze training and test sessions) are summarized in Supplementary Table 1.

### Behavioral training

#### Rewarded task

This task is described in detail in Martinez et al. (2022). Briefly, rats were placed in a behavioral operant chamber containing a “nose-poke” at which they could seek the reward by licking through a slot onto a lick tube. Breaking an infrared beam in front of the nose-poke ended the inter-trial interval (ITI), and 200 ms after, the nose-poke was then illuminated for 100 ms (visual cue). Following the visual cue, licks were detected by breaking a second infrared beam. Upon licking the required number of times (8 licks), a reward (∼20 μL of water) was made available in half of the trials on a pseudo-random fashion. Water reward was delivered through a tube with a solenoid attached to it. The behavioral task was controlled and registered using an Arduino Uno board. Trials ended when the animal removed its head from the nose-poke. Subjects were trained with one session per day, that ended when they completed 300 trials or reached a maximum training time of 120 min. As all the animals were trained for 15 sessions, we defined “early” and “late” as the first 3 and the last 3 training sessions, respectively, similar to the method applied by Zold and Hussain Shuler (2015). Timely trials are those in which the subject entered the nose-poke after the required minimum waiting time. Premature trials are those in which the entrance beam was interrupted before reaching the minimum waiting time. All behavioral data are expressed as mean ± SEM (except for reward rate figures, where the median is indicated, and the Waiting time curves, where the accumulated frequency of trials for each bin is shown, from all the rats in the group). Each dot corresponds to the average value of a session from a single animal.

#### Open field

The apparatus is a 50 cm wide × 50 cm long × 40 cm-high arena with gray wooden walls and floor, divided into 9 squares by blue lines. Rats were introduced to the arena individually and left to explore it for 10 min. We registered the number of crossings between squares and the number of rearings. Twenty-four hours later, to test the animals’ habituation memory, they were re-introduced in the arena for 10 min and crossings and rearings were registered. In parallel, we recorded the sessions using a webcam above the maze. The videos were used to quantify the time spent in the central square in the training session using Bonsai software (Lopes et al., 2015). Before each exploration session, the arena was cleaned with a mixture of 70:29.5:0.5 alcohol: water: detergent to remove olfactory cues.

#### Object recognition in the Y-maze

This paradigm is based on the animals’ natural tendency to explore novel objects or places. Object recognition was conducted in a white Y-shaped acrylic maze (de Landeta et al., 2020). The central arm of the maze was 27 cm long and 10 cm wide, and the short arms were 8.5 cm long and 10 cm wide. All the outer walls were 40 cm high to prevent the animal from visualizing any external cues. In the habituation session, rats were allowed to explore the empty maze for 10 min for 1 day. During the sample phase (Training, Tr), rats were placed in the central arm and left to explore for 5 min two identical objects placed in each of the short arms of the apparatus. The objects used here were similar in size and height and of different colors and textures: a plastic cup upside down placed over a soda can or a glass jar of instant coffee. Animals were actively exploring when they sniffed or touched objects with their paws. On the contrary, trying to stand over an object or biting was not considered exploration. The Choice phase (Test, Ts) was conducted 24 h after the sample phase and rats were left to explore two different objects for 3 min, one familiar and the other novel.

The Novel Object *Preference Index* (Novel Object discrimination index) was calculated as (*T*_*novel*_−*T*_*familiar*_)/(*T*_*novel*_ + *T*_*familiar*_), where where T_*novel*_ and T_*familiar*_ correspond to the exploration time spent on the novel and familiar objects, respectively. Positive values indicate preference for the novel object, whereas values not significantly different from zero indicate no preference (i.e., absence of recognition memory). The maze and objects were thoroughly cleaned with 70% ethanol-detergent solution between sessions.

Behavioral testing included both the Open Field and Object Recognition Y-maze tasks; while the Open Field generally preceded the Y-maze, the order was reversed in a small subset of adults (females, *n* = 4; males, *n* = 2) due to logistical constraints related to shared equipment. Data is detailed in Supplementary Table 1.

### Quantification and statistical analysis

#### Behavioral analysis

**FIGURE 1:**
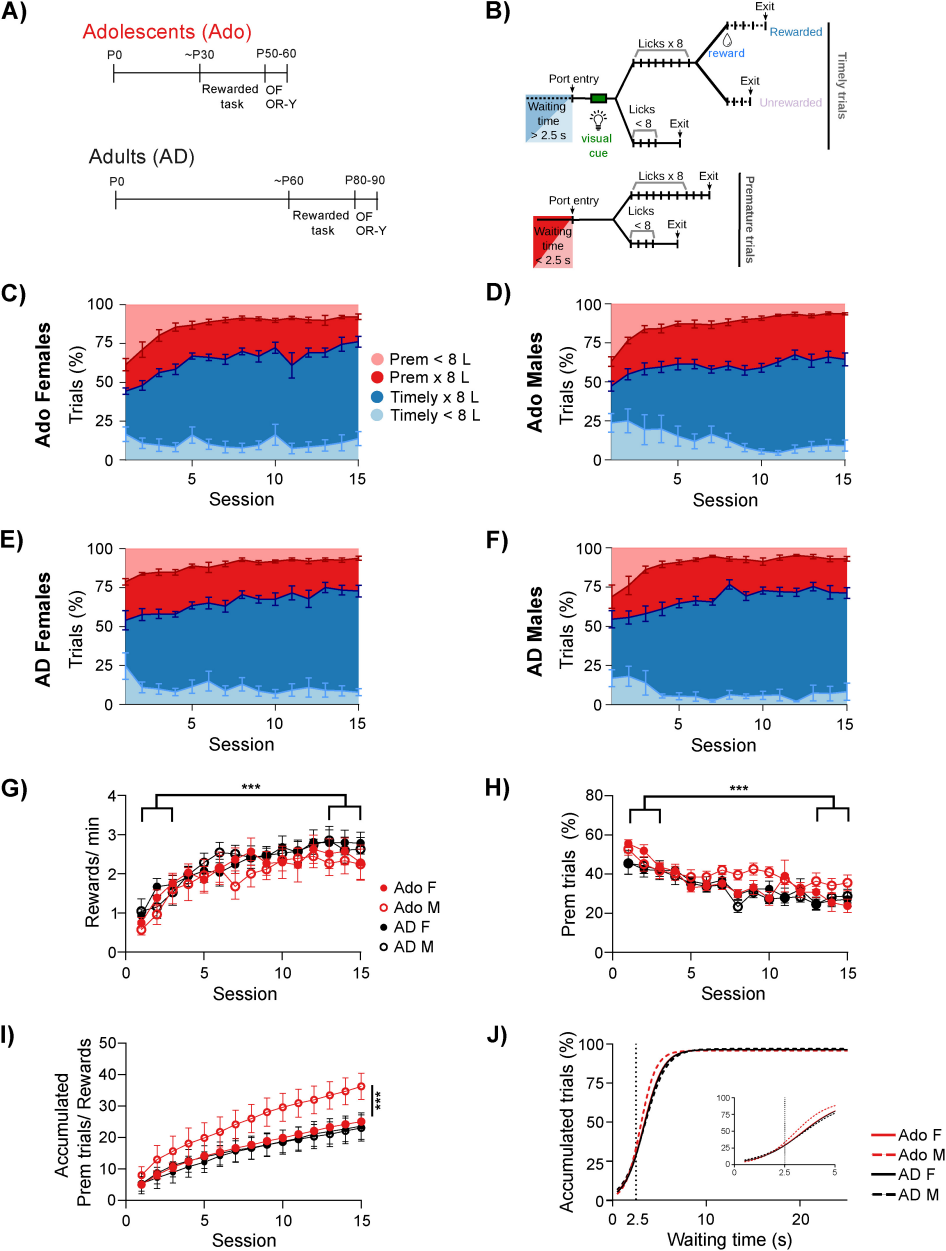
Adolescent and Adult animals of both sexes become skilled in the rewarded task. **(A)** Experimental timeline. Animals were trained for 15 daily sessions in the rewarded task followed by testing in the Open Field (OF) and Object Recognition in the Y-maze (OR-Y). Training start ages were as follows: Adolescents (∼P30): 8 females (P31–36) and 8 males (P28–36). Adults (∼P60): 8 females (P59–70) and 8 males (P60–72). **(B)** Description of the different trial types. *Timely trials* are those in which the animal waits > 2.5 s before entering the nose-poke and initiating the licking sequence; half of these trials are rewarded. *Premature trials* occur when waiting time is < 2.5 s and are never rewarded. Diagram adapted from Martinez et al. (2022), eLife, under a Creative Commons Attribution (CC BY) license. **(C,D)** Learning curves of adolescent females **(C)** and males **(D)**. **(E,F)** Learning curves of adult females **(E)** and males **(F)**. **(G)** Reward rate per session. **(H)** Percentage of premature trials per session. **(I)** Accumulated premature trials per number of obtained rewards. **(J)** Logistic fits of accumulated trials per session for different waiting times; inset, zoom around the 2.5-s threshold. **(C–F)** Data are expressed as mean ± SEM. **(G–I)** Data are expressed as mean ± SEM; females are represented with filled circles and males with open circles. Adolescents (Ado) are shown in red and Adults (AD) in black. **(J)** Adolescents (Ado) are shown in red and Adults (AD) in black, males are depicted with dashed lines. **(G,H)** ***p < 0.0001, Early vs. Late, Three-way Repeated Measures ANOVA (time factor); **(I)** ***p < 0.0001, Linear Regression model.

Data curation and calculation of session-level measures (e.g., number of trials of each type, mean number of licks, latency to the first lick, 8-lick sequence or trial duration, waiting times, and generation of the accumulated-trials curves shown in Figure 1J) were performed in MATLAB using custom-built scripts. MATLAB was also used for the regression analysis shown in Figure 1I. All remaining statistical analyses were conducted in Prism 10 (GraphPad Software). Figures were generated using colorblind-safe palettes from ColorBrewer 2.0.

All data was assessed for normality. Because one group did not meet normality assumptions (adolescent males), we used a Kruskal–Wallis test to compare the center occupancy for the four groups. All comparisons (early vs. late) × age × sex, (training vs. test) × age × sex, (first vs. last minute) × age × sex, were made with repeated measures three-way ANOVA. Comparisons for age, sex, training and trial type were made with multiple way ANOVA in MATLAB. One-way ANOVA and one sample *t*-test were used to analyze the Preference Index in Object recognition. A general linear model was used to compare accumulated premature responses from adult and adolescent rats (Figure 1I). Model: response ∼1+ trial × age + trial × sex + age × sex. Accumulated trials for the different waiting times (Figure 1J) were fitted with a logistic function (y ∼ p1/(1 + exp (−p2 *(x − p3)))). Coefficient p1 is related to the bottom part of the curve, coefficient p3 is related to the top part of the curve, and coefficient p2 is related to the slope, where higher values describe steeper curves.

The scripts used for these procedures have been deposited alongside the dataset on OSF and are available as Supplementary material.

## Results

### Adolescent and Adult rats learn the rewarded task

We trained water-deprived adolescent and adult rats (Figure 1A) in the rewarded task previously described in Martinez et al. (2022). After a visual cue, rats could self-initiate a trial and obtain a water reward by completing an 8-lick sequence at the reward spout. If animals started a trial > 2.5 s after the end of the previous one, the trial was considered “Timely.” This gave them visual feedback that reported a 0.5 probability of receiving a water reward. In contrast, prematurely initiated trials (<2.5 s waiting time) were penalized by re-initiating the timer and did not have a visual cue associated with them (Figure 1B).

Learning curves of adolescent females (Figure 1C, mean age at the beginning of the experiment = 34.75) and adolescent males (Figure 1D, mean age at the beginning of the experiment = 30.75) trained for 15 consecutive sessions show that both groups learned to make timely entries followed by the 8-lick sequence. Like adult females (Figure 1E, mean age at the beginning of the experiment = 67.25) and adult males (Figure 1F, mean age at the beginning of the experiment = 68.75, Figure 1D), adolescents improved their performance with training, increasing the reward rate. A three-way mixed ANOVA with Time as a repeated factor confirmed a robust main effect of time *F*_(1, 28)_ = 81.95, *p* < 0.001, partial η^2^ 0.745 (Figure 1G). Following our previous findings, adolescents initially made more premature responses than adults, but toward the final training sessions their behavior became comparable to the older group, showing fewer premature trials and more complete lick sequences. A three-way mixed ANOVA with Time as a repeated factor analysis on the evolution of premature responses across sessions again revealed a significant effect of Time, *F*_(1, 28)_ = 58.43, *p* < 0.001, partial η^2^ = 0.676 (Figure 1H).

We analyzed performance *within* the different trial types. All groups showed comparable latencies to initiate the 8-lick sequence. The four-way mixed ANOVA revealed significant main effects of trial type [*F*_(2, 408)_ = 7.47, *p* = 0.0007, partial η^2^ = 0.035] and training stage [*F*_(1, 408)_ = 45.02, *p* < 0.0001, partial η^2^ = 0.099]. In contrast, sex [*F*_(1, 408)_ = 3.39, *p* = 0.066, partial η^2^ = 0.008], age [*F*_(1, 408)_ = 0.16, *p* = 0.693, partial η^2^ = 0.0004], and all interactions were not significant (Supplementary Figure 1A).

The time to complete the lick sequence was influenced by sex, age, and training stage. Females completed the sequence faster overall. Animals improved their performance from early to late sessions [*F*_(1, 528)_ = 97.07, *p* < 0.0001, partial η^2^ = 0.155]. Trial type had no effect, and most interactions were nonsignificant, except for a modest Age × Stage interaction [*F*_(1, 528)_ = 6.26, *p* = 0.013, partial η^2^ = 0.012], reflecting different improvement trajectories in adolescents and adults (Supplementary Figure 1B).

For the total time required to complete each trial, performance varied across trial types, ages, and training stages. The ANOVA showed significant main effects of trial type [*F*_(2, 552)_ = 81.00, *p* < 0.0001, partial η^2^ = 0.227], age [*F*_(1, 552)_ = 19.23, *p* < 0.0001, partial η^2^ = 0.034], and stage [*F*_(1, 552)_ = 147.85, *p* < 0.0001, partial η^2^ = 0.211]. Sex had no significant effect [*F*_(1, 552)_ = 2.03, *p* = 0.155, partial η^2^ = 0.004], and no Trial interactions were detected. The only significant interaction was Age × Stage [*F*_(1, 552)_ = 7.13, *p* = 0.0078, partial η^2^ = 0.013], again reflecting age-dependent performance improvement across stages.

Licking variability (Licks CV) decreased markedly across training and was lower in adults than adolescents. The ANOVA revealed significant main effects of age [*F*_(1, 552)_ = 24.75, *p* < 0.0001, partial η^2^ = 0.043] and stage [(*F*_(1, 552)_ = 63.84, *p* < 0.0001, partial η^2^ = 0.104]. Trial type [*F*_(2, 552)_ = 2.37, *p* = 0.094, partial η^2^ = 0.008] and sex [*F*_(1, 552)_ = 0.79, *p* = 0.375, partial η^2^ = 0.001] were not significant. Most interactions were nonsignificant, except for a Sex × Stage interaction [*F*_(1, 552)_ = 9.71, *p* = 0.0019], indicating that males and females differed in how their variability changed across training. All the results for these parameters of the performance in the trials are summarized in Supplementary Figure 1E. Taken together, age and training stage shaped how animals executed the trials, but by late training most measures converged across groups.

Although adult males and females performed similarly, adolescent males showed a markedly distinct profile. Throughout training adolescent male rats accumulated significantly more premature trials than adolescent females despite earning the same number of rewards (Figure 1I). A linear regression including trial number, age, sex, and their interactions fit the data significantly better than a constant model [*R*^2^ = 0.376, *F*_(47.6, 473)_ = 47.6, *p* < 0.001]. Trial progression had the strongest effect [*t*_(473)_ = 8.874, *p* < 0.001, partial η^2^ = 0.143], followed by a smaller but significant contribution of sex [*t*_(473)_ = 3.215, *p* = 0.001, partial η^2^ = 0.021], reflecting elevated premature responding in adolescent males. Age alone did not contribute [*t*_(473)_ = 0.999, *p* = 0.318, partial η^2^ = 0.002]. The trial × age interaction was significant but small [*t*_(473)_ = −2.026, *p* = 0.043, partial η^2^ = 0.009]. Finally, there was a significant age × sex interaction [*t*_(473)_ = −4.409, *p* < 0.001, partial η^2^ = 0.039] supporting the distinct behavioral profile of adolescent males.

Furthermore, consistent with our previous observations, the logistic fit of accumulated trials across waiting times for the late sessions in training showed a markedly steeper growth in adolescent males (Figure 1J). This was reflected in the *p2* coefficient of the logistic function (profile-likelihood 95% CI): adolescent males = 1.222 (1.139–1.313), adolescent females = 0.9681 (0.9122–1.029), adult females = 0.9674 (0.9161–1.022), and adult males = 0.4525 (0.8153–0.9266), with a significant main effect of group [*F*_(3, 1,172)_ = 19.07, *p* < 0.001]. For example, at 3.2 s waiting-time, adolescent males had accumulated 50% of the trials, whereas the other groups had reached a 10% less. A similar pattern is observed near the 2.5-s limit, providing further evidence that adolescent males initiate trials more impulsively than the other groups (Figure 1J, inset).

### Increased impulsivity is not explained by locomotor activity

**FIGURE 2:**
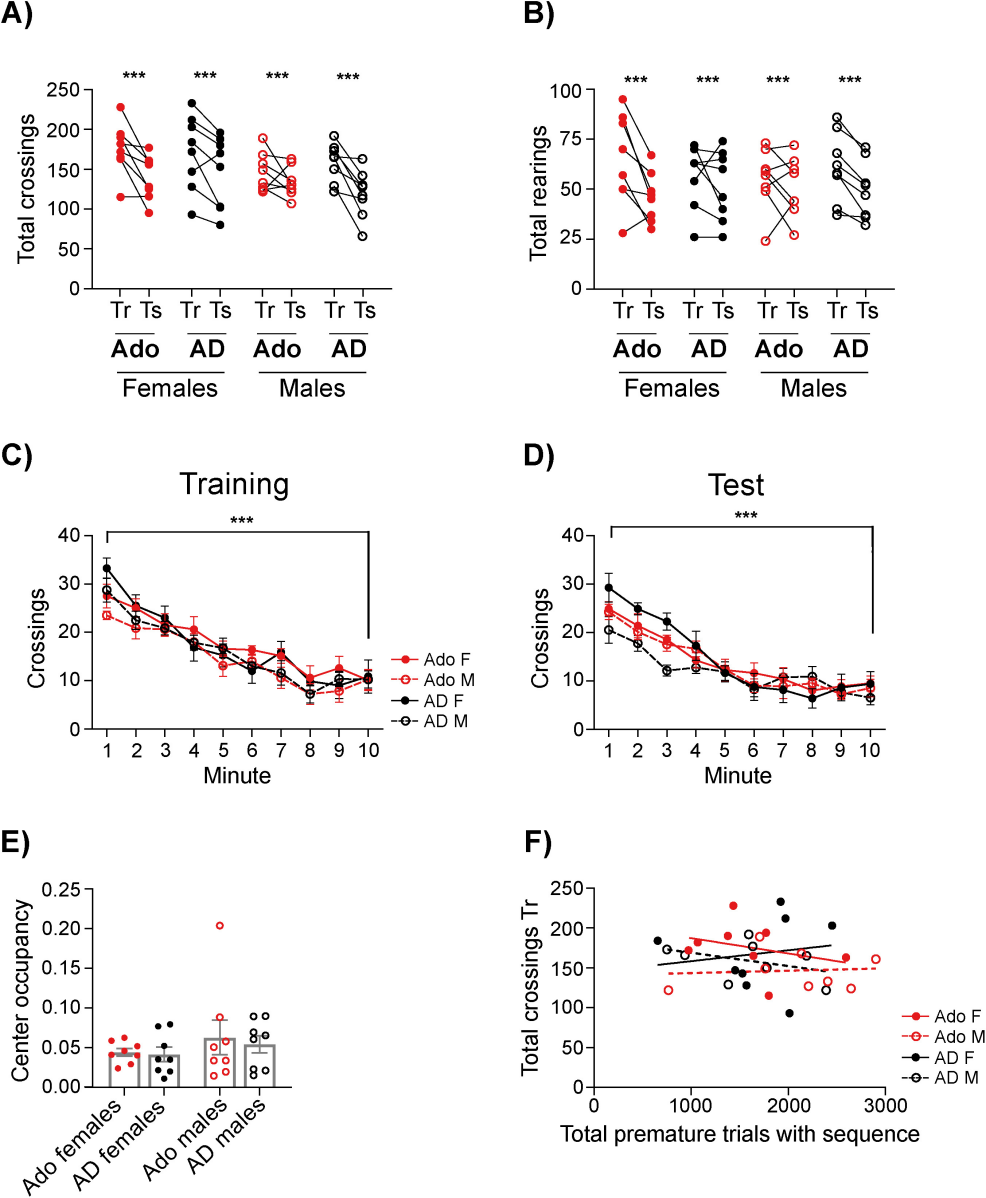
Performance in the OF. Number of crossings **(A)** and rearings **(B)** in the training and test sessions of all groups. **(C,D)** Habituation within session, expressed as a reduction in the crossings- during training **(C)** and test **(D)** sessions. **(E)** Proportion of the time spent in the central square of the arena. Data in **(A,B)** are expressed as total number of events per session; data in **(C–E)** are shown as mean ± SEM. Females are represented with filled circles, males with open circles; Adolescent (Ado) are shown in red and Adults (AD) in black. **(A)** ***p < 0.0001 for the time factor, Three-way Repeated Measures ANOVA; **(B)** *p* < 0.0001 for the time factor, Three-way Repeated Measures ANOVA. **(C,D)** ***p < 0.001 first vs. last minute, Minute × Age × Sex Three-way ANOVA, repeated factor: minute. **(E)** Kruskal-Wallis test (NS). **(F)** Relationship between the total number of premature trials accumulated across all training sessions and total crossings during the training session of the open field. Each point represents one animal. Lines indicate least-squares fits shown for visualization purposes. Pearson correlation analyses revealed no significant associations in any group (NS).

We tested the performance of the rats in the Open Field. Both adolescents and adults exhibited long-term memory for the arena as the number of crossings and rearings was significantly less in the test session [Figure 2A, crossings: Crossings, Time, *F*_(1, 28)_ = 36.99, *p* < 0.0001, partial η^2^ = 0.569; Time × Sex × Age, *F*_(1, 28)_ = 5.468, *p* = 0.0267, partial η^2^ = 0.163, Three-way ANOVA with Time as the repeated factor]. During training (Tr) and testing (Ts), average crossings (mean ± SEM) were as follows: adolescent females (Tr: 176.1 ± 11.41; Ts: 139.6 ± 9.82), adolescent males (Tr: 146.6 ± 8.61; Ts: 133.9 ± 6.66), adult females (Tr: 167.9 ± 16.9; Ts: 146.5 ± 15.9), and adult males (Tr: 159.4 ± 8.4; Ts: 119.0 ± 10.5).

A similar effect was observed for rearings [Figure 2B; Time, *F*_(1,__28)_ = 16.37, *p* = 0.0004, partial η^2^ = 0.368], and exploratory activity was generally comparable between sexes. Within each session, all groups also displayed a similar habituation profile. During training, adolescents and adults showed a significant reduction in the number of crossings [Figure 2C, first vs. last minute: *F*_(1, 28)_ = 134.3, *p* < 0.001, partial η^2^ = 0.827] and rearings [data not shown, first vs. last minute, *F*_(1, 28)_ = 63.73, *p* < 0.001, partial η^2^ = 0.694] using a Three-way ANOVA with minute as the repeated factor. Likewise, in the test session, there also was a significant reduction in the number of crossings [Figure 2D, first vs. last minute, *F*_(1, 28)_ = 165.9, *p* < 0.001, partial η^2^ = 0.856] and rearings [data not shown, first vs. last minute, *F*_(1, 28)_ = 30.11, *p* < 0.001, partial η^2^ = 0.518, Three-way ANOVA with minute as the repeated factor] across the first and last minutes for all groups.

Because adolescence is associated with increased risk-taking, we also examined the time spent in the central square of the arena in the training session, when exploratory or anxiolytic behavior should be more overt than in the test. As one group (adolescent males) did not meet normality assumptions, we used a Kruskal–Wallis test, which revealed no group differences [Figure 2E, *H*_(3)_ = 0.991, *p* = 0.803].

Finally, to determine whether locomotor activity was related to impulsive behavior, we performed Pearson correlation analyses, which revealed no significant association between the total number of premature trials accumulated across all training sessions and the number of crossings in the training session of the Open Field in any group (Figure 2F; adolescent females: *r* = −0.30, 95% CI [−0.83, 0.51]; adolescent males: *r* = 0.08, 95% CI [−0.66, 0.74]; adult females: *r* = 0.15, 95% CI [−0.62, 0.77]; adult males: *r* = −0.39, 95% CI [−0.86, 0.43]; all *p*s > 0.33).

Together, these findings indicate that although sex and age influence some aspects of locomotor activity, these differences are insufficient to explain the increased impulsivity observed in adolescents.

### Adolescents and adults show a similar recognition memory

**FIGURE 3:**
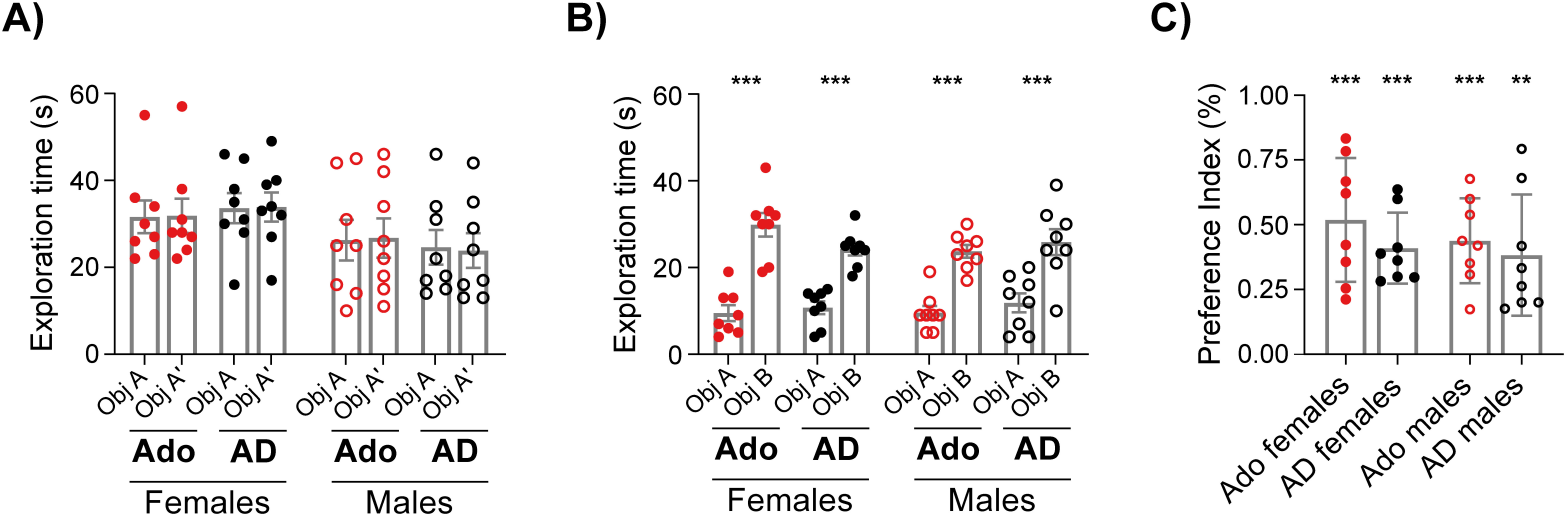
Object recognition in the Y-OR. **(A)** Total exploration time of the identical objects (A–A’) during the training session. **(B)** Total exploration time of the objects (A,B) during the test session. **(C)** Preference Index for each group during the test. Data are expressed as mean ± SEM. Females are represented with filled circles and males with open circles; adolescents (Ado) in red and adults (AD) in black. **(A)** Three-way ANOVA: no effects of object, sex, or age. **(B)** ***p < 0.0001 (object factor), Three-way ANOVA with object as the repeated factor. **(C)** One-way ANOVA across groups: n.s.; one-sample *t*-tests vs. chance (0): **p < 0.01, ***p < 0.001.

Next, we tested whether adolescents presented a similar strategy and long-term memory for object recognition in the Y-maze. Before the beginning of the experiments, because it was uncertain whether adolescents and adults would differ in their locomotor activity, we selected the paradigm used by de Landeta et al. (2020). This task has the advantage of testing recognition memory but not being as dependent on locomotor activity as spatial object recognition (Ballarini et al., 2009). In the training session, the total time the animals spent exploring a pair of identical objects did not differ by object, sex or age [Figure 3A, object: *F*_(1,__28)_ = 0.012, *p* = 0.9134, partial η^2^ = 0.0004, sex: *F*_(1,__28)_ = 3.491, *p* = 0.0722, partial η^2^ = 0.111, age: *F*_(1,__28)_ = 0.001, *p* = 0.9750, partial η^2^ = 3.58 × 10^–5^, three-way ANOVA with Object as the repeated factor]. In the test session, behavior was also similar between groups [Figure 3B, object: *F*_(1,__28)_ = 151.7, *p* < 0.0001, partial η^2^ = 0.844, sex: *F*_(1, 28)_ = 0.2533, *p* = 0.6187, partial η^2^ = 0.009, age: *F*_(1, 28)_ = 0.000, *p* > 0.9999, partial η^2^ = 0, three-way ANOVA with Object as the repeated factor]. All groups exhibited robust object recognition memory, evidenced by the Preference Index [Figure 3C, *F*(_3,__28)_ = 0.701, *p* = 0.559, partial η^2^ = 0.069, One-way ANOVA]. One-sample t-tests against chance (0) revealed significant preference indexes in all groups [Adolescent females: *t*_(7)_ = 6.135, *p* = 0.0005, η^2^ = 0.843, Adult females: *t*_(7)_ = 8.463, *p* < 0.0001,η^2^ = 0.911, Adolescent males: *t*_(7)_ = 7.558, *p* = 0.0001, η^2^ = 0.891, Adult males: *t*_(7)_ = 4.625, *p* = 0.0024, η^2^ = 0.753].

## Discussion

Impulsivity is a complex and multifaceted construct that is profoundly influenced by the neurodevelopmental changes that occur during adolescence. Here, we show that male adolescent rats exhibit significantly more premature responses in a rewarded operant task than females. These impulsive actions can be characterized as *waiting impulsivity*, in which animals are unable to withhold responding until the inter-trial interval (ITI) has elapsed, and as *stopping impulsivity*, which typically refers to the inability to suppress an initiated action (Dalley and Ersche, 2019), reflected by the tendency to initiate and carry out the action sequence to completion even when it is not rewarded. Notably, premature responding was not significantly associated with Open Field locomotor activity or with measures of cognitive performance, indicating that the observed sex differences in impulsive behavior were not readily explained by differences in general locomotion or cognition.

Adolescence is a period of rapid developmental change, and even small age differences can reflect meaningful shifts in gonadal hormones (Bell, 2018). In our study, males began training slightly earlier (P28–36) than females (P31–36). This resulted in males performing the initial training mostly before pubertal onset, whereas females trained during early puberty. This modest age difference could introduce some degree of variability. However, we prioritized matching the length of training over strict age-matching (as an exploratory extension of previous work), to compare how learning evolved across ages. Importantly, the rewarded task spanned 3–4 weeks of training, so all animals—regardless of starting age—completed their training well after the onset of puberty. The pattern we observed reflects stable behavioral tendencies expressed over the course of development. We also did not observe age-related differences in the OF and Y-maze—conducted near the end of adolescence—, and a complementary cohort tested specifically for age effects showed comparable results, and females did not display greater variability in any control measures despite potential hormonal fluctuations. Taken together, these considerations indicate that age-related factors are unlikely to fully explain the elevated premature responding observed in adolescent males.

Recent evidence indicates that locomotor responses to novelty are not static across development and may not stabilize as a trait until late adolescence (approximately postnatal day 58 (Kirschmann et al., 2025). Accordingly, OF testing in our adolescent cohort spanned postnatal days 38–57, a transitional period during which these motor behaviors are still maturing. Importantly, our primary findings on premature responding remained significant when postnatal age was accounted for, supporting the conclusion that the observed impulsivity differences are not attributable to developmental shifts in general exploratory drive.

More broadly, adolescents are known to exhibit heightened reward sensitivity and reduced sensitivity to costs relative to adults, predisposing them to impulsive, reward-driven behaviors (Spear, 2000; Laviola et al., 2003; Stolyarova and Izquierdo, 2015; Klune et al., 2021). Consistent with this, adolescent males made more premature responses to obtain the same number of rewards as adults, suggesting a hyperactive or heightened engagement in reward-driven actions, in line with prior work showing that adolescent males exert greater effort toward rewards and are less sensitive to extinction (Andrzejewski et al., 2011; Stolyarova and Izquierdo, 2015). Using the same task, we have previously found that dorsal striatum activity correlates with premature responding and anticipatory behavior in adolescent males (Martinez et al., 2022).

Sex differences during adolescence likely reflect distinct developmental and hormonal trajectories. Puberty occurs earlier in females, whereas males undergo a more protracted period in which inhibitory control systems—including white matter reorganization—continue to mature (Silveri et al., 2006; Bava and Tapert, 2010). Hormones such as estradiol modulate dopaminergic signaling and time perception, producing sex-specific effects that vary with developmental stage and hormonal state (Colzato et al., 2010; Pleil et al., 2011). In rodents and humans, females show cycle-dependent fluctuations in inhibitory control and learning (Bohacek and Daniel, 2010; Colzato et al., 2010; Bayless et al., 2012; Hamson et al., 2016; Ladouceur et al., 2019). Although estrous cycle monitoring was beyond the scope of the present study, females did not show greater variability in any control measures; even so, cycle-related influences cannot be ruled out.

These developmental differences may also influence motivation and action timing. Studies have reported sex-dependent differences in how animals acquire or maintain reward-guided strategies, with females often showing more consistent performance across sessions (Bayless et al., 2012; Panfil et al., 2020; Peck et al., 2020, 2022; Chen et al., 2021). Although our study was not designed to examine underlying mechanisms, the pattern of results is consistent with the idea that sex-dependent maturation of cognitive control contributes to the elevated premature responding observed in adolescent males.

Importantly, this interpretation aligns with task- and context-dependent patterns reported in other paradigms. In the 5CSRTT, for example, adult males exhibit more premature responses under unpredictable cue conditions, whereas females tend to acquire task strategies more rapidly and respond more consistently (Bayless et al., 2012; Chen et al., 2021). Male rats also show a stronger preference for smaller-sooner over larger-later rewards and are more responsive to delay-exposure training than females (Panfil et al., 2020; Peck et al., 2020, 2022). Likewise, stimulant–reward interactions can selectively increase vulnerability in adolescent males (Ruiz et al., 2018). Together, these findings suggest that males tend to exhibit higher behavioral variability, whereas females show greater stability in reward-guided decision making.

Finally, premature responses of adolescent males are unlikely to reflect general increases in locomotion or exploration, as we observed no differences across groups in an exploratory task (OF) or in recognition memory (Y-maze). This aligns with evidence that impulsive action is dissociable from exploratory activity (Arrondeau et al., 2023) or other behavioral traits, such as risk-related decision-making (Urueña-Méndez et al., 2024), suggesting that impulsive action may reflect partially distinct underlying processes.

In summary, our results indicate that adolescent males show greater impulsive responding than females in a reward-based operant task, independent of exploratory, anxiety-like behaviors or cognitive performance. This sex-specific phenotype likely reflects differences in the maturation of cortico-striatal networks, modulated by hormonal influences (Weafer and de Wit, 2014; Hammerslag et al., 2019; Klune et al., 2021). These findings extend evidence from human studies, where males are generally more prone to sensation-seeking behaviors while inhibitory control tends to be stronger in females (Cross et al., 2011; Shulman et al., 2015; Hammerslag and Gulley, 2016). Given the established association between impulsivity and vulnerability to addiction and psychiatric disorders (Weafer and de Wit, 2014; Dalley and Ersche, 2019), understanding its developmental and sex-specific underlying mechanisms may provide valuable insights into the origins of maladaptive behaviors and target prevention strategies for at-risk populations.

## Data Availability

The datasets presented in this study can be found in online repositories. The names of the repository/repositories and accession number(s) can be found at: https://osf.io/4nwjv.
