# Supplementary material for: Premature release of action sequences in adolescent male rats
Source: Front Behav Neurosci. 2026 Feb 10;20:1716850. doi: 10.3389/fnbeh.2026.1716850 (PMC12929139; doi:10.3389/fnbeh.2026.1716850)
Supplement: Supplementary file 1 [file Image_1.pdf]

Supplementary Table 1)

Litter ID	DOB	Sex	N	Age group	RT start age (d)	OF training age (d)	Y-maze training age (d)
1	7/2/2021	F	2	AD	59	81	79
2	10/4/2021	M	2	AD	60	86	89
3	30/5/2021	F	2	Ado	36	38	60
		M	2	Ado	36	38	60
4	2/9/2021	F	4	AD	68	84	89
5	23/2/2022	M	4	Ado	28	54	56
		F	2	AD	70	82	78
		M	2	AD	70	82	78
6	2/5/2022	F	4	Ado	35	57	65
		M	4	AD	72	94	99
7	13/8/2022	M	2	Ado	31	52	60
		F	2	Ado	31	52	60

Values indicate age in postnatal days. Open Field and Y-maze tests were conducted 24 h after their respective training sessions.

Supplementary Figure 1

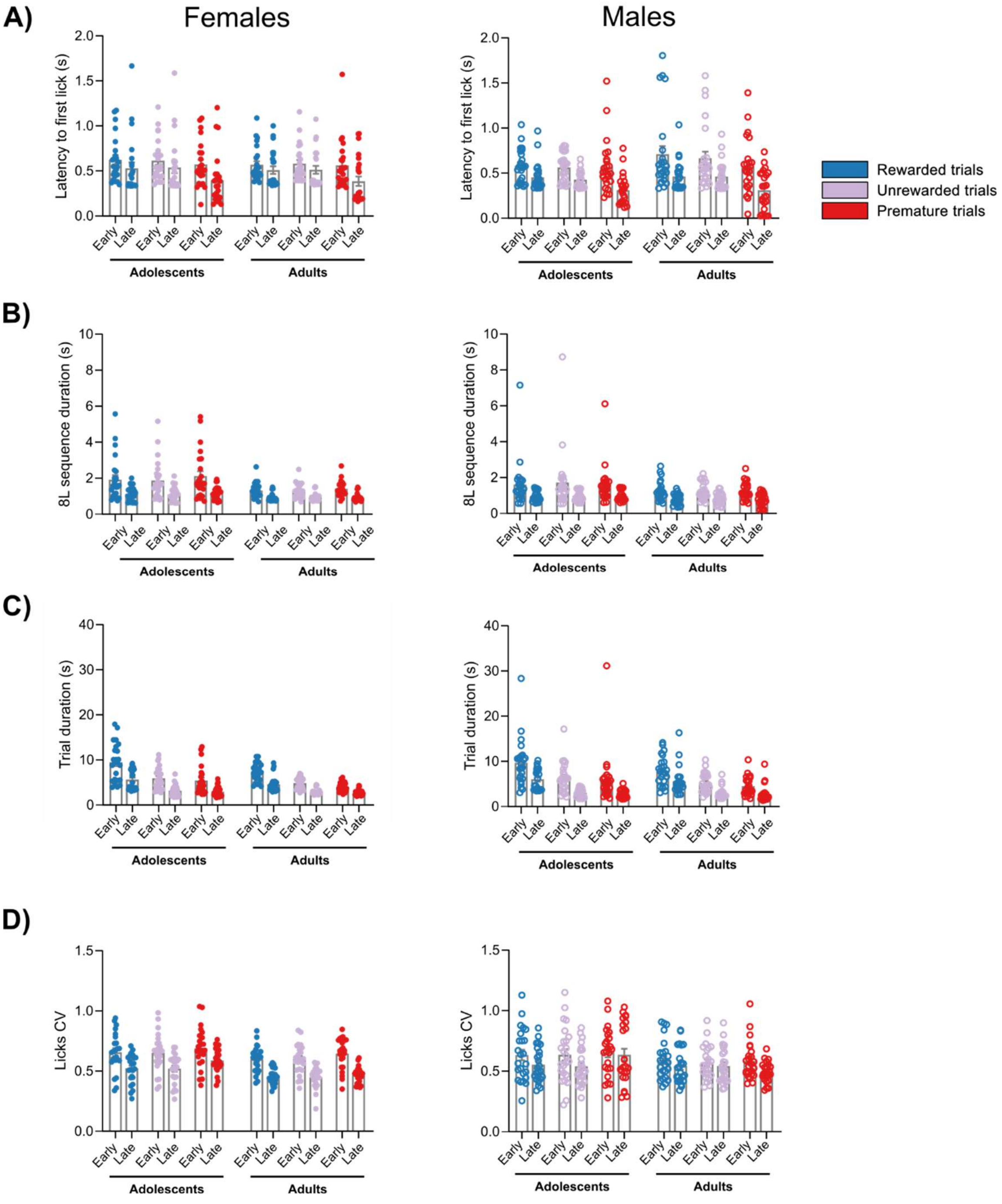

E)

Variable	Trial type	Stage	Age	Sex	Interactions
**Latency to first lick**	$F_{(2,408)}$=7.47, p=0.0007, η^2=0.035	$F_{(1,408)}$=45.02, p<0.0001, η^2=0.099	NS	NS	NS
**8L sequence duration**	NS	$F_{(1,528)}$=97.07, p<0.0001, η^2=0.155	$F_{(1,528)}$=34.16, p<0.0001, η^2=0.061	$F_{(1,528)}$=6.01, p=0.0145, η^2=0.011	Age × Stage: $F_{(1,528)}$=6.26, p=0.0126, η^2=0.012
**Trial duration**	$F_{(2,552)}$=81.00, p<0.0001, η^2=0.227	$F_{(1,552)}$=147.85, p<0.0001, η^2=0.211	$F_{(1,552)}$=19.23, p<0.0001, η^2=0.034	NS	Age × Stage: $F_{(1,552)}$=7.13, p=0.0078, η^2=0.013
**Licks CV**	NS	$F_{(1,552)}$=63.84, p<0.0001, η^2=0.104	$F_{(1,552)}$=24.75, p<0.0001, η^2=0.043	NS	Sex × Stage: $F_{(1,552)}$=9.71, p=0.0019, η^2=0.017

Supplementary Figure 1. Performance across ages, sexes, trial types, and training stages. Symbols represent the average value within each training session. Females are shown with filled circles and males with open circles. Data are expressed as mean ± SEM for Early and Late sessions (three sessions per stage were included for each animal).

A) Latency to initiate the 8-lick sequence was comparable across groups. A four-way mixed ANOVA revealed significant main effects of trial type and training stage, with no effects of sex or age, and no significant interactions.

B) Time to complete the lick sequence varied with sex, age, and training stage. Females completed the sequence faster overall, and all groups improved from Early to Late sessions. Trial type had no effect; a modest Age × Stage interaction indicated different improvement trajectories between adolescents and adults.

C) Total trial duration showed significant main effects of trial type, age, and training stage, with no effect of sex and no trial-related interactions. An Age × Stage interaction again reflected age-dependent performance improvement across stages.

D) Licking variability (CV) decreased across training and was overall lower in adults than adolescents. Significant main effects of age and stage were observed, along with a Sex × Stage interaction indicating that males and females differed in how their variability changed across training.

E) Summary of statistically significant main effects and interactions from four-way mixed ANOVAs for each behavioral variable. For each significant effect, F values, p values, and partial eta squared (η^2) are reported; non-significant effects are indicated as "NS".
